# Implant Surface Decontamination Methods That Can Impact Implant Wettability

**DOI:** 10.3390/ma17246249

**Published:** 2024-12-20

**Authors:** Georgios E. Romanos, Lauren Mistretta, Allyson Newman, Danielle Ohana, Rafael A. Delgado-Ruiz

**Affiliations:** 1 Laboratory of Periodontal-, Implant-, Phototherapy, Department of Periodontics and Endodontics, Stony Brook University, Stony Brook, NY 11794, USA; lauren.mistretta@stonybrookmedicine.edu (L.M.); allyson.newman@stonybrookmedicine.edu (A.N.); danielle.ohana@stonybrookmedicine.edu (D.O.); 2 Department of Prosthodontics and Digital Technology, School of Dental Medicine, Stony Brook University, Stony Brook, NY 11794, USA; rafael.delgado-ruiz@stonybrookmedicine.edu

**Keywords:** air-polishing, decontamination, lasers, wettability

## Abstract

This review addresses the effects of various decontamination methods on the wettability of titanium and zirconia dental implants. Despite extensive research on surface wettability, there is still a significant gap in understanding how different decontamination techniques impact the inherent wettability of these surfaces. Although the literature presents inconsistent findings on the efficacy of decontamination methods such as lasers, air-polishing, UV light, and chemical treatments, the reviewed studies suggest that decontamination alters in vitro hydrophilicity. Post-decontamination surface chemistry must be carefully considered when selecting optimal surface treatments for implant materials. Further in vitro investigations are essential to determine which approaches best enhance surface wettability, potentially leading to improved implant–tissue interactions in clinical settings.

## 1. Introduction

The histological evaluation of hydrophilic surfaces has confirmed early osseointegration [1]. Surface wettability affects the behavior of adhesion proteins, interactions between hard- and soft-tissue cells with conditioned surfaces, bacterial adhesion, biofilm formation, and the rate of osseointegration [2]. It also plays a significant role in the healing and regeneration of tissues surrounding the implant surface [3]. Peri-implantitis lesions do not always respond to treatment in a predictable manner, and therefore the wettability of the implant surface in the defect area is fundamental from a clinical perspective. Wettability is influenced by the surface topography, roughness, and chemical composition; thus, any method that alters these characteristics can modify surface wettability [4,5,6]. Increasing surface roughness appears to enhance wettability, while chemical composition changes can either increase or decrease wettability, depending on the treatment applied [4,5,6]. The wettability of a surface is indicated by its water contact angle: angles between 0° and 90° indicate hydrophilic surfaces, while angles greater than 90° indicate hydrophobic surfaces. Hydrophilic surfaces are known to preserve the conformation and function of proteins, while hydrophobic textures can lead to protein denaturation due to conformational changes. Protein adsorption significantly influences the ability of cells to attach to and migrate across implant surfaces, with hydrophilic surfaces demonstrating a greater affinity for proteins compared with hydrophobic ones. Furthermore, a higher degree of hydrophilicity is associated with the enhanced differentiation and maturation of osteoblasts, which can accelerate the process of osseointegration [4].

Factors such as surface topography, roughness and wettability, which influence implant osseointegration, can be related directly to the implant design and surface treatment. Le Guéhennec et al. examined various techniques to increase surface roughness and to apply osteoconductive coatings to titanium dental implants [5]. Surface treatments, such as titanium plasma spraying, grit blasting, acid-etching, anodization, and the application of calcium phosphate coatings, along with their associated surface morphologies and properties, were reviewed. Most of these treatment methods are commercially available and have demonstrated clinical efficacies exceeding 95% over a five-year period. There have also been various decontamination methods explored for zirconia implants, such as acid-etching, laser irradiation, UV treatment, and the application of different coatings [6].

Mechanical debridement methods have shown more favorable results compared with laser and chemical treatment methods [7]. Of the mechanical debridement techniques, air-polishing has been shown to have the best cleaning effectiveness compared with other methods [7]. However, conflicting data suggest that air–powder abrasion with glycine powder and 3 W diode laser irradiation have a minimal impact on the physicochemical properties of implant surfaces compared with other methods [8].

Although numerous studies have investigated surface treatments for decontaminating dental implants, their specific effects on surface wettability remain unclear. This review addresses this gap by examining various physical and chemical decontamination methods, with a focused analysis on how each technique influences surface wettability (Table 1).

## 2. Implant Surface Decontamination Methods

Decontamination of implant surfaces is essential to reduce the effects of bacteria and their subproducts on the peri-implant tissues. There are three methods for decontaminating implant surfaces—physical, mechanical, and chemical—with each having unique mechanisms of action that address the bacterial colonization of implant surfaces [9,10].

Mechanical methods, while discussed in the literature, were purposefully excluded from this review due to their tendency to not only alter the chemical properties of the implant surface but to also significantly modify the implant’s topography. By focusing solely on physical and chemical approaches, this review aims to provide a comprehensive overview of methods that maintain implant integrity while effectively addressing microbial contamination.

### 2.1. Physical Decontamination Methods

The physical decontamination methods discussed in this review include the use of lasers, air-polishing, UV light, and cold atmospheric plasma. We first outline the mechanism of action for each method, followed by an analysis of its impact on surface wettability.

#### 2.1.1. Surface Decontamination with Lasers

Among the various methods for decontaminating an implant surface, laser irradiation has many advantages, such as high reproducibility, the ability to control surface textures, and the alteration of surface properties. In terms of surface properties, laser irradiation has been shown to enhance the wettability, roughness, and morphology and to format different surface textures (nano and micro textures) [11]. The erbium:yttrium–aluminum–garnet (Er:YAG) laser has become an effective instrument for surface decontamination. Er:YAG lasers have been shown to cause less of a temperature increase and fewer surface alterations to titanium surfaces compared with other types of lasers, such as the neodymium-doped yttrium, aluminum, and garnet or diode lasers [12]. Findings by Friedmann et al. suggest that the Er:YAG laser was effective in allowing increased attachment of osteoblast cells after the removal of *P. gingivalis* and cell compounds [13]. Romanos et al. showed that laser irradiation of titanium surfaces with carbon dioxide or Er,Cr:YSGG lasers may promote osteoblast attachment and osteoblastic proliferation [14].

Ayobian-Markazi et al. analyzed the effects of Er:YAG laser irradiation on SLA titanium disks and evaluated their biocompatibility [15]. The results indicated that using an Er:YAG laser with a 100 mJ energy intensity is safe for titanium decontamination, improving the biocompatibility of the disks and increasing osteoblast responsiveness. In addition, an in vitro study investigated the physicochemical changes in titanium disks (SLA) contaminated with *Escherichia coli* after treatment with Er:YAG irradiation lasers and air-flow abrasion [16]. There was no significant difference in the contact-angle measurements of the disks after Er:YAG irradiation versus air-flow abrasion; however, there was a significant difference between the experimental disks compared with the control as the laser-irradiated and air-flow disks had lower contact angles than the untreated disks. SEM analysis also indicated no difference between the laser-irradiated and air-flow-treated disks. Additionally, both the air-flow-treated and laser-treated groups showed a notable decrease in the carbon percentage, indicating a likely reduction in surface contamination from hydrocarbons. Although the air-flow abrasion group’s carbon percentage was marginally lower than that of the erbium group, the difference was not statistically significant. These findings suggest that the Er:YAG laser, operating at a wavelength of 2940 nm and energy of 150 mJ/pulse, does not produce carbonation or harmful side effects on the surface of SLA disks.

An in vitro study examined the impact of decontamination methods on surface characteristics, including the topography, wettability, chemical composition, and biocompatibility of titanium implant surfaces previously contaminated with biofilms [17]. There were two types of titanium disk surfaces: micro-rough (sandblasted, large-grit, acid-etched; SLA) and smooth (machined; M). Microcosm biofilms were cultivated on both SLA and M Ti disks, which were subsequently treated using various protocols: Ti brushes (TiB) alone, a combination of TiB and photodynamic therapy (PeriowaveTM; Vancouver, BC, Canada (λ: 660–675 nm; 11 mW)), TiB with 0.2% CHX/1% NaClO, and treatments with or without ultraviolet-C (UV-C) radiation. None of the treatment methods hindered the biocompatibility of the titanium surface; however, chemical agents and micro-rough surfaces rendered a greater cytotoxic effect in MG-63 cells [17].

Rezeka et al. evaluated the effect of diode laser irradiation on the hydrophilicity, surface topography, and chemical composition of SLA titanium disks [18]. Group I consisted of control disks that were not subjected to laser irradiation. Group II included disks irradiated with a diode laser at a wavelength of 940 nm (Epic X; Biolase) at a power setting of 1 W. Group III disks were irradiated at a power of 2 W, while Group IV disks received irradiation at a power of 3 W. All other parameters were standardized across the groups. The disks were irradiated using a continuous laser beam for a duration of 30 s. The wettability was measured using the sessile drop method by a contact-angle goniometer after diode laser treatment, and the contact angle significantly decreased on the treated disks compared with the non-treated disks. The largest contact angle was measured in the control group (90.5 ± 14.5°), and the lowest contact angle was measured in the 3 W group (55.7 ± 17.4°). The surface topography was observed, and wider grooves, greater pores, and wider melting foci were observed in the 2 W group. The complete disfigurement of surface details with wide zones were imaged in the 3 W group, and the 1 W group’s topographical appearance did not differ from that of the control disks [18].

#### Impact of Lasers on Surface Wettability

Laser irradiation is capable of promoting surface changes in titanium and zirconia surfaces, including to the surface roughness, topographic elements, and wettability. Al-Khafaji and Hamad conducted a comprehensive evaluation of commercially pure titanium disks that were laser-structured using two distinct designs: dot and groove [19]. Each design was subjected to three varying laser scan speeds (5, 15, and 25 mm/s) and compared against control titanium surfaces that underwent no structuring. The study focused on measuring both the wettability and the surface roughness to determine the effects of these treatments. A contact-angle measuring device (Creating Nano Technologies Inc., Taiwan) was employed to assess the surface wettability of the titanium disks. The findings revealed that laser structuring significantly enhanced the wettability of the titanium surfaces, with the groove design demonstrating particularly advantageous results across all scan speeds. Notably, as the number of laser scans increased, the surface roughness also increased, resulting in a decrease in the water contact angle—indicating improved wettability of the commercially pure titanium disks. This decrease in contact angle with increasing laser scans may be attributed to the enhanced crevice formation on the titanium surface, leading to greater roughness in both experimental designs.

In a study investigating the effect of the Er:YAG laser on the properties of sandblasted and acid-etched (SLA) titanium disks, it was found that the laser irradiation decreased the surface roughness and increased the wettability of twenty-one SLA titanium surfaces [15]. Contact angles were measured after distilled water was deposited on the disk surfaces. The median contact angles for the non-treated control disks were 133.4° (more hydrophobic), and the experimental disks had a median contact angle of 111.9° (more hydrophilic). Laser irradiation of the SLA disks decreased the surface roughness as well. The study by Khosroshahi et al. supported these findings, as laser irradiation with the Nd:YAG laser at 140 J/cm^2^ improved the SaOs-2 cell viability on titanium surfaces (Ti6Al4V), which resulted from increased hydrophilicity of the implant surface [20]. Lee et al. investigated the effect of different irradiation methods (Er,Cr:YSGG lasers and diode lasers) on different Grade 4 titanium disk surfaces: sandblasted, large-grit, acid-etched, and femtosecond laser-treated [21]. Wettability was measured using a contact-angle goniometer with drops of deionized water as the liquid medium. On femtosecond-laser-treated titanium implant surfaces, Er,Cr:YSGG and diode laser irradiation significantly reduced the surface contact angle, enhancing wettability (control = 82.2°; diode laser = 74.3°; Er,Cr:YSGG laser = 73.8°; electrocautery = 76.2°; *p* = 0.039). However, machined and SLA disks had an insignificant change in surface wettability after laser irradiation. Due to the disparity in data, more in vitro studies need to be conducted to further determine the influence of lasers on the wettability of dental implants. Contact-angle analysis of titanium implant surfaces after Er:YAG laser irradiation at lower energy levels should be a future direction of research.

Staehlke et al. analyzed yttria-stabilized zirconia disks that were micro-structured with the laser micro processing system RDX1000, creating various different geometric micro-patterns [22]. The water contact angle was measured with the sessile drop method using the Drop Shape Analyzer. The micro-rough surfaces produced by the laser process demonstrated a decrease in surface wettability (Table 2).

#### 2.1.2. Surface Decontamination with Air-Polishing

Microbial contamination of implant surfaces has been shown to inhibit the formation of new osseous tissues as well as playing a key role in the initiation of peri-implant diseases [23,24]. Mombelli and Lang emphasized that to effectively treat peri-implantitis, one must thoroughly decontaminate the implant surface, which involves the eradication of bacteria and toxins as well as the removal of both supragingival and subgingival biofilms [25]. As an alternative to mechanical debridement, air-polishing has been introduced to clean implant surfaces, reducing the biofilm. In titanium disks treated by air-polishing with glycine powder, the surface roughness increased, and the amount of residual biofilm was 8.6-fold lower than in the control group [24].

**Figure 1:**
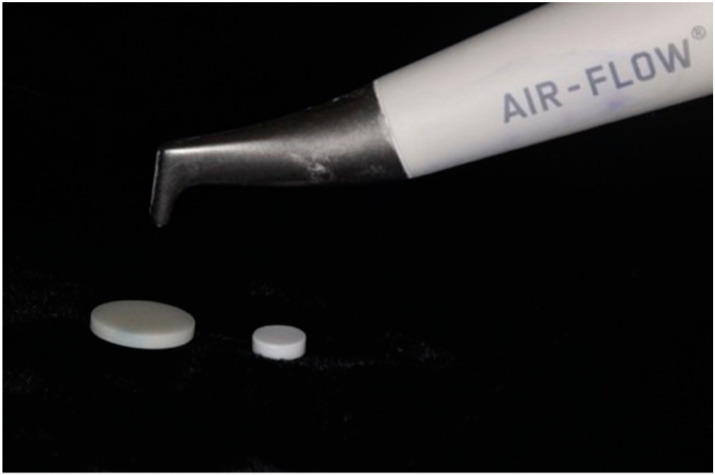
Air-polishing of two zirconia disks using the Airflow^®^ handpiece with Air-flow^®^ (Nozzle EMS, Nyon, Switzerland). Handpiece held at a 90-degree angle and 5 mm distance from the disks [29].

Air-polishing (Figure 1) has been shown to reduce biofilms on periodontally compromised teeth and implant surfaces and is comparable to conventional biofilm removal methods such as ultrasonics and curettes. During air-polishing therapy, abrasion from the low molecular weight powder, water, and pressurized air removes the biofilm from the surface. There are many different options for powders, and studies have shown that glycine powder and erythritol powder are the least damaging to surfaces [26]. The particle size of glycine is 4× smaller than that of sodium bicarbonate particles [27], and in a study comparing air-polishing treatment with glycine powder to sodium bicarbonate powder, the use of glycine caused less damage to the titanium implant surface while reducing bacterial recolonization when compared with titanium surfaces using sodium bicarbonate [28].

Francis et al. compared the effects of six different air-polishing powders, sodium bicarbonate (65 mm and 40 mm), glycine, erythritol (+/− cetylpyridinium chloride), and calcium carbonate on titanium disks [30]. The different treatments elicited an 89.3% and 88.6% biofilm reduction for *S. sanguinis* on moderately rough and machined disks, respectively. All the powders showed statistically significant superiority for biofilm removal compared with calcium carbonate powder. In an additional study, biofilm removal was analyzed on sandblasted titanium disks after two different air-polishing treatments: glycine powder or erythritol–chlorhexidine powder. Although both glycine and erythritol–chlorhexidine displayed inhibitory activity against bacterial strains (*S. aureus*, *B. fragilis* and *C. albicans*), the erythritol–chlorhexidine powder was more effective in biofilm removal [31]. It was demonstrated in a rabbit tibia model that a decontamination method using air-polishing therapy with a chlorhexidine rinse and an Er:YAG laser increased biofilm removal from and osseointegration onto titanium disks [32].

A case report showed that air-polishing with erythritol powder combined with guided tissue regeneration is successful in the short-term clinically and radiographically, achieving adequate re-osseointegration [33]. Furthermore, in a randomized controlled clinical trial, implants were randomly treated by air-polishing with erythritol or saline-soaked cotton gauzes during resective surgery. When looking at parameters such as bleeding on probing, the, plaque score, probing depth, marginal bone loss, periodontal full-mouth scores and levels of eight classical periodontal pathogens, it was found that there was no significant difference between the effectiveness of air-polishing with erythritol powder compared with saline surface cleansing when looking at the previously mentioned clinical parameters. Toma et al. conducted a clinical evaluation of three different surgical modalities—plastic curettes, air-polishing with glycine powder, and a rotating titanium brush—for peri-implantitis treatment [34]. After being evaluated at three different time points (baseline, three months post-surgery and six months post-surgery), it was found that the titanium brush and the glycine air-polishing device were more effective for peri-implantitis treatment than the use of plastic curettes. Air-polishing with erythritol powder was shown to be just as effective as piezoelectric ultrasonic scaling in peri-implantitis treatment [35].

#### Air-Polishing and Surface Wettability

In addition to removing the biofilm, air-polishing has the ability to change the surface properties of implant surfaces. There is a limited amount of literature on the impact of air-polishing on implant surface wettability. Huang et al. evaluated the surface changes and bacterial adhesion on titanium and zirconia disks after different decontamination procedures, which included titanium curette treatment, carbon-fiber-reinforced plastic curette treatment, ultrasonic scaling with carbon-fiber-tip treatment, air polishing with glycine powder, and a control group [36]. After instrumentation, the arithmetical mean roughness (Ra), hydrophilicity, and surface free energy were recorded. A contact-angle goniometer was used with deionized water droplets utilizing the sessile drop method. The results indicated that zirconia disks displayed a higher wettability than corresponding titanium disks with the same decontamination method. However, the air-polishing treatment showed reduced hydrophilicity compared with the control groups for both groups (*p* < 0.05). Wettability assessment from an in vitro evaluation of peri-implantitis modalities on Saos-2 osteoblasts included the treatment of titanium disks with a plastic curette, air-polishing, a titanium brush, or implantoplasty [37]. This in vitro study showed that the control-, plastic-curette-, Perio-Flow- and Ti-Brush-treated surfaces were hydrophobic, with a significant decrease in the surface wettability of the plastic curette disks.

An in vitro study with conflicting results analyzed the wettability of titanium disks via the water contact-angle method after a seven-day biofilm was removed [38]. The disks were either treated by air-polishing with erythritol powder, cold atmospheric pressure, or both methods. The water contact angle was measured in the sessile drop mode and was shown to be reduced by all the treatment methods, exhibiting an increased surface wettability. Kister et al. evaluated the influence of peri-implantitis coating instruments on the integrity of photoactive nanocoating [39]. Anatase-coated titanium disks were treated with diamond burs, polishers, plastic and metal hand instruments, an air scaler, and air-flow devices. An air-flow device (KaVo PROPHYflex^®^ 3) in combination with a sodium bicarbonate powder (ProphyPowder, KaVo) was applied from a distance of 0.5 cm for 20 s. Only those surfaces treated with the air scaler, the air-polishing device, and ProCup reached a superhydrophilic state with contact angles below the threshold of 10°, while the surfaces treated with plastic and metal curettes showed a total decrease of more than 30° but without reaching a superhydrophilic state. Another in vitro study analyzed the effect of air-polishing with glycine or erythritol powders on different zirconia disks. The wettability was measured via a contact-angle goniometer (sessile drop technique) after four different liquids—saline, bovine serum albumin (BSA), bovine thrombin (BioPharm), and bovine artificial blood (LAMPIRE Biological Lab)—were dropped onto the center of each disk. Erythritol had a greater increase in wettability than glycine. It was shown that erythritol increased the surface wettability of zirconia disks significantly better than glycine air-polishing [29] (Table 3).

#### 2.1.3. Surface Decontamination with UV Light

Another approach to dental implant surface modification involves ultraviolet (UV) light irradiation, which has been shown to enhance the wettability of certain material surfaces, thereby improving their biological activity. UV treatment is a simple, low-cost method that has proven effects for many types of tested titanium surfaces [40]. However, direct exposure to UV radiation after dental implant placement can have harmful effects, as UV-A light (320–400 nm) may trigger skin-damaging effects such as carcinogenesis [41]. To mitigate these risks, UV-light pretreatment before implant placement can be utilized to enhance surface properties and to avoid damage to human cells.

UV-light irradiation has emerged as a promising method for enhancing the surface properties of dental implants. UV photofunctionalization, or the treatment of titanium with UV light, is now an established method for increasing the bioactivity and osseointegration of titanium implants [42]. While many studies have shown the biological and clinical outcomes of photofunctionalization, there questions still remain about the optimal treatment duration and the extent of its maximum efficacy.

Over time, titanium implants accumulate atmospheric hydrocarbons, creating a layer that reduces the surface wettability and rendering the titanium hydrophobic [43]. This negatively impacts the recruitment, attachment, and proliferation of osteogenic cells and hinders bone–titanium integration. UV photofunctionalization enhances osteoconductivity by decarbonizing titanium surfaces, thereby restoring the bioactive properties and improving implant integration with bone tissue [44]. Additionally, photocatalytic activity on titanium surfaces can promote antimicrobial function that may help to prevent the development of peri-implant mucositis, a known risk factor for the osseointegration process in the early healing stage [45].

#### UV Light and Surface Wettability

The first study to report an increase in the hydrophilicity of titanium dioxide (TiO_2_) crystals by UV irradiation was published by Wang et al. [46]. Using both anatase and rutile TiO_2_ surfaces, they found that UV irradiation generates oxygen vacancies at bridging oxygen sites on the surface, causing the conversion of Ti^4+^ sites to Ti^3+^ sites. This conversion due to UV irradiation makes the TiO_2_ surface more favorable for dissociative water adsorption.

In an in vitro study by Rupp et al., thin pulse-sputtered anatase layers were deposited onto grade 2 commercially pure titanium (cp Ti) disks [47]. After UV irradiation with an intensity of 25 mW/cm^2^ for 60 and 120 s, the contact angles on cp Ti control disks without anatase did not decrease to below 58°, even with a UV dosage of 346 J/cm^2^. On the other hand, the anatase films showed super hydrophilic behavior, with contact angles of <5° after UV treatment for a minimum of 75 s at a minimum dosage of 1.9 J/cm^2^. This UV-induced super hydrophilization caused water droplets to spread immediately on anatase films on Ti, showing promise for the use of UV treatment in improving the osseointegration of anatase-coated implant surfaces.

Another study by Park et al. compared the surface characteristics of anodized titanium disks (control group) with UV-irradiated anodized titanium disks (test group) [48]. Both the control and test groups were anodized under 300 V, and then the test group was irradiated by a 15 W bactericidal UV lamp for 24 h before the experiment. Using the sessile drop method, images were obtained after dropping 3.4 μℓ of water onto six disks per group for contact-angle measurements. Five points in each disk (30 points total for each group) were used for the contact-angle analysis. The results showed a statistically significant difference (*p* < 0.001) between the control and test groups. The UV-irradiated disks were found to be more hydrophilic than the anodized disks without further surface modification, with contact-angle measurements of 22.69 ± 4.35° (mean ± SD) for the test group and 77.43 ± 3.80° (mean ± SD) for the control group. This significant decrease in contact angle supports the findings from Rupp et al. [47], highlighting the potential for UV light treatment to enhance interactions between anodized titanium implant surfaces and the biological environment.

In addition to titanium, zirconia (ZrO_2_) is a frequently used implant material and is favorable for implants placed in the oral esthetic zone [49]. Watanabe et al. (2012) used tetragonal zirconia polycrystal (TPZ) disks that were UV-treated for 2 h with UV radiation at a power of 19 mW/cm^2^ [50]. Excitation wavelengths of 185 nm and 254 nm corresponded to UV-C light, while 365 nm corresponded to UV-A light. Contact-angle measurements were made at three different locations for each disk 3 s after a 4 μL water droplet was applied to the disk surfaces. The results showed that the control disks had a mean contact angle of 78.7 ± 8.3°, while the UV-treated disks showed a significant decrease to a superhydrophilic contact-angle measurement of 0°.

A study by Tuna et al. tested two different zirconia-based implant disks (Zr1 and Zr2) with either smooth or roughened surfaces [51]. Zr1 consisted of oxides of metals including aluminum, zirconium, yttrium, cerium, hafnium, and magnesium. Zr2 consisted of a more conventional yttrium tetragonally stabilized zirconium oxide with zirconium, hafnium, and yttrium. The disks were treated by UV light for 15 min, which was delivered as a mixture of spectra at wavelengths of 360 nm and 250 nm via a single UV lamp source. One μℓ droplets of water were used for contact-angle analysis at four different areas on a total of 34 disks. The results showed that for all the groups, UV treatment caused a significant increase in hydrophilicity (*p* ≤ 0.0001) from a hydrophobic to a hydrophilic state.

A more recent study on zirconia-based materials found no significant difference between zirconia treated with and without UV-C light [52]. High-translucent (ZrO-HT) and ultra-translucent multi-layered (ZrO-UTML) zirconia disks were used. Ten disks per group were randomly selected for UV-C light treatment with a peak wavelength of 253.7 nm for 48 h. For the contact-angle analysis, five specimens were measured from each group, with the results showing no significant differences in hydrophilicity between the treated and untreated disks.

#### 2.1.4. Plasma Surface Decontamination

Plasma is a state of matter in which an ionized gas is electrically neutral. It consists of a complex combination of ions, electrons, neutral particles, UV photons, and free radicals [53]. When the electron temperature within this ionized gas significantly exceeds the bulk plasma temperature, this is termed “cold atmospheric plasma” [54]. Cold atmospheric plasma (CAP) is recognized for its non-thermal properties and can be generated using various gases, including helium, argon, nitrogen, heliox (a blend of helium and oxygen), and air. Notably, the temperature of CAP at the point of application remains below 104 °F, making it suitable for sensitive biological surfaces [55].

CAP is gaining recognition as an innovative treatment method for peri-implantitis due to its dual capabilities in antimicrobial action and surface modification [56]. The technique shows promise in enhancing the removal of bacterial plaque from dental implant surfaces, especially in challenging anatomical areas or during surgical interventions such as open-flap debridement. By effectively targeting biofilms, CAP can facilitate the re-integration of compromised dental implants and enhance overall treatment outcomes [57].

The mechanism by which CAP exerts its antimicrobial effects involves the generation of charged particles and reactive oxygen and nitrogen species (RONS). These RONS have been shown to penetrate bacterial cell walls and disrupt cellular integrity, leading to microbe inactivation. Specifically, the oxidative stress induced by RONS can cause damage to vital cellular components, including proteins, lipids, and nucleic acids, ultimately resulting in cell death [56]. This property makes CAP a valuable adjunctive therapy in the management of peri-implantitis, as it not only aids in the decontamination of infected surfaces but may also promote a favorable healing environment by reducing the bacterial load [58].

The application of CAP has been shown to positively influence the wettability of implant surfaces, an important factor for osseointegration and the overall success of dental implants. The favorable changes in wettability achieved through plasma treatment can enhance protein adsorption and cell adhesion, which are critical for the regeneration of healthy tissue around implants [59].

#### Plasma Decontamination and Surface Wettability

In a study by Hui et al., the researchers aimed to compare the efficacy of two treatment modalities: air abrasion using erythritol powder and CAP in a liquid medium, both separately and in combination. The study utilized saliva from a peri-implantitis patient to develop biofilms on 35 moderately rough titanium implants. The results indicated the successful growth of a complex human biofilm, with all treatment modalities demonstrating significant decontamination effects on the implant surfaces. Specifically, the air-abrasion treatment alone and the combined air-abrasion and CAP treatment showed remarkable reductions in biofilm presence, with removal percentages nearing that of the negative control (94.87% and 95.32%, respectively). Notably, CAP alone yielded a lower removal rate of 52.10%. SEM analysis revealed no significant alterations in the titanium surface features across all the treatment groups, indicating that the treatments preserved the implant’s topography. The study concluded that the combination of air abrasion and CAP effectively removes biofilms from titanium implants without compromising their surface characteristics. However, it noted that the observed effects of CAP alone were minimal, suggesting the need for further investigation into its potential as a standalone treatment or its synergistic effects with other decontamination methods [60].

Another study by Flörke et al. assessed the efficacy of three adjunctive therapy options for decontaminating titanium implant surfaces: cold atmospheric plasma (CAP), photodynamic therapy (PDT), and chemical decontamination using 35% phosphoric acid gel (PAG). The implants were placed in a simulated bone defect, contaminated with *Enterococcus faecalis*, and then treated with one of the three modalities. The study found that CAP treatment resulted in a mean of 1.24 × 10^5^ CFU/mL, significantly lower than that of the PDT group, which had 8.28 × 10^6^ CFU/mL, and the PAG group, with 3.14 × 10^6^ CFU/mL. The CAP-treated implants exhibited the highest reduction in live bacteria, demonstrating its superior antimicrobial effect compared with both PDT and PAG. The authors concluded that while none of the treatment options achieved complete decontamination of the titanium surface, CAP showed promising results as an adjunctive therapy alongside mechanical debridement for managing peri-implantitis due to its effectiveness in reducing bacterial colonization [61].

In a more recent study by Martins et al., the authors investigated the effects of CAP treatment on titanium surfaces enclosed in surgical-grade packaging, with the aim of enhancing the hemocompatibility and bacterial resistance of dental implants. The titanium disks were subjected to CAP treatment, resulting in a significant reduction in the contact angle. The results indicated that CAP treatment significantly improved the wettability of titanium surfaces, as evidenced by a lower contact angle (14 ± 2.2°) compared with that of untreated samples (59 ± 1.8°), while preserving the crystalline structure of the titanium. The CAP-treated surfaces exhibited enhanced hemocompatibility, demonstrated by faster clot formation in prothrombin time (PT) and activated partial thromboplastin time (APTT) tests compared with polished controls. Additionally, the study found that the CAP-treated samples showed increased platelet activation, density, and thrombus formation, suggesting improved interaction with blood components. Microbiological analyses further revealed that the CAP treatment effectively reduced bacterial colony formation by *Pseudomonas aeruginosa*, highlighting its potential as a method for decontaminating dental implants while enhancing their biocompatibility [54] (Table 4).

### 2.2. Chemical Treatment Decontamination Methods

The chemical treatment decontamination methods discussed in this review include chlorhexidine (CHX), hydrogen peroxide (H_2_O_2_), citric acid, ethylenediaminetetraacetic acid (EDTA), and sodium hypochlorite (NaOCl). We first outline the general mechanism of action for the chemical followed by an analysis of its impact on surface wettability.

#### 2.2.1. Chlorhexidine (CHX) Decontamination

Chlorhexidine (CHX) is a widely used antimicrobial agent known for its broad-spectrum efficacy against both Gram-positive and Gram-negative bacteria as well as some fungi. Its mechanism of action on implant surfaces involves disrupting the integrity of the bacterial cell membrane. CHX is a cationic bisbiguanide compound, meaning that it carries a positive charge that allows it to bind to the negatively charged bacterial cell wall [62]. This interaction leads to increased permeability of the cell membrane, causing the leakage of intracellular components such as potassium and phosphorus, which are essential for cellular functions [63]. Different concentrations of CHX are commonly used in concentrations ranging from 0.1% to 2% for implant surface decontamination:

1. 0.1% to 0.2% CHX—Lower concentrations, often used as mouth rinses or irrigation solutions, are less aggressive on tissues and can be effective for maintaining peri-implant health. For instance, 0.2% CHX has been used as an irrigation solution in cases of peri-implant mucositis to reduce the bacterial load [64].
2. 1% CHX gel—This concentration is commonly applied directly to implant surfaces during non-surgical peri-implantitis treatments. It allows for localized application, reducing bacterial colonization while minimizing tissue irritation. Studies indicate that 1% CHX gel may be effective in reducing microbial contamination on implants [65].
3. 2% CHX solution—Higher concentrations, such as 2%, are typically used in clinical settings for mechanical debridement or adjunctive surgical treatments of peri-implantitis. The 2% solution provides potent antimicrobial action, which is particularly useful when a more aggressive approach to decontamination is required [66].

At low concentrations, CHX can be bacteriostatic, inhibiting bacterial growth by interfering with essential cellular processes. At higher concentrations, CHX becomes bactericidal, meaning that it kills bacteria outright. This is achieved by causing irreversible damage to the cell membrane, leading to cell lysis and death. In dental implants, CHX is applied to reduce the microbial load, particularly in cases of peri-implantitis, where bacterial colonization on the implant surface can lead to inflammation and bone loss. Due to its substantivity, CHX binds to proteins in the oral environment, allowing for prolonged antimicrobial effects even after the initial application [62,63,64,65,66].

#### Chlorhexidine (CHX) Decontamination and Surface Wettability

Research on the effect of CHX on implant surface wettability has yielded mixed results. Stuani et al. found that CHX application on dental implant surfaces did not significantly alter wettability in vitro, although they observed residual CHX and its constituents adhering to the titanium surface even after rinsing. This residual presence suggests that CHX has a strong affinity for titanium surfaces, likely due to its substantivity, which allows it to remain on surfaces for extended periods [67]. In contrast, Baryak et al. reported improved wettability on titanium SLA (sandblasted, large-grit, acid-etched) surfaces treated with CHX, as evidenced by lower contact angles. They attributed this increased wettability to the CHX residue being adsorbed on the surface, which may alter the surface energy and enhance hydrophilicity [68]. The variability observed is probably related to the concentration and type of CHX used in these studies.

#### 2.2.2. Hydrogen Peroxide (H_2_O_2_) Decontamination

Hydrogen peroxide (H_2_O_2_) is an effective chemical agent used for the decontamination of dental implant surfaces due to its strong oxidative properties. Its mechanism of action involves the production of reactive oxygen species (ROS), which have powerful antimicrobial effects on a wide range of microorganisms, including bacteria commonly associated with peri-implantitis. Hydrogen peroxide (H_2_O_2_) acts on implant surfaces through three primary mechanisms: (1) the production of reactive oxygen species (ROS), which damages bacterial cell walls, leading to cell death; (2) biofilm disruption, where ROS break down biofilm structures, making bacterial eradication more effective and reducing recolonization; and (3) surface cleaning and oxide layer formation, where H_2_O_2_ removes organic residues and forms a stable oxide layer on titanium, enhancing surface stability and potentially improving osseointegration [9].

Different concentrations of H_2_O_2_ have been used for the decontamination of implant surfaces, including H_2_O_2_ 3% and H_2_O_2_ 6%. The surfaces were exposed from 1 to 3 min, achieving almost complete decontamination of the implant surfaces [69,70].

#### Hydrogen Peroxide (H_2_O_2_) Decontamination and Surface Wettability

The oxidation process induced by H_2_O_2_ appears to enhance the surface characteristics of titanium implants by increasing the wettability, protein adsorption, and cell proliferation. Mouhyi et al. demonstrated that H_2_O_2_ treatment combined with a carbon laser restored the oxide layer and improved wettability on contaminated titanium implant surfaces [71]. Similarly, Yoneyama et al. found that a 3% H_2_O_2_ hydrothermal treatment for three minutes significantly reduced the water contact angle on titanium surfaces, indicating enhanced wettability [72]. Furthermore, brushing implant surfaces with 3% H_2_O_2_ not only inhibited bacterial regrowth but also increased the surface energy of previously contaminated titanium implants [73].

However, the effects of H_2_O_2_ on zirconia surfaces are inconsistent. Noro et al. reported no change in zirconia wettability after H_2_O_2_ treatment [74]. In contrast, Tuna et al. found that a 20% H_2_O_2_ treatment for two hours increased the surface wettability of zirconia but also raised the monoclinic phase content, heightening the risk of thermal degradation of zirconia polycrystals [75].

#### 2.2.3. Citric Acid Decontamination

Citric acid is used to decontaminate titanium implant surfaces due to its acidic properties and its ability to effectively disrupt biofilms. Its mechanism of action involves several key processes. First, citric acid breaks down the extracellular matrix of biofilms, facilitating easier removal of bacterial colonies from the implant surface. It also functions as a chelating agent by binding to essential metal ions, such as calcium and magnesium, in bacterial cell walls, which weakens the biofilm structure and increases bacterial susceptibility. Additionally, citric acid helps remove endotoxins and organic and inorganic contaminants, resulting in a cleaner surface. Furthermore, citric acid may enhance the surface energy of titanium by modifying the oxide layer, potentially improving the implant’s compatibility with the surrounding tissue. However, high concentrations of citric acid or residual traces left on the implant can negatively impact bone cell attachment to the treated surfaces [76,77].

#### Citric Acid Decontamination and Surface Wettability

An in vitro study by Wheelis et al. found that citric acid decontamination led to noticeable changes on the implant surface as observed by scanning electron microscopy [78]. Specifically, the treatment increased surface roughness as the citric acid dissolved the oxide layer originally present on the implant disk. In contrast, a study by Souza et al. demonstrated that citric acid effectively decontaminated the surface without affecting the electrochemical stability of titanium, indicating that citric acid’s impact may vary based on specific treatment conditions [79]. The effect of citric acid on the surface properties of implant surfaces, and thereby the re-osseointegration process, is still unclear.

#### 2.2.4. Ethylenediaminetetraacetic Acid (EDTA) Decontamination

Data regarding the effect of EDTA on implant surface properties and re-osseointegration is very limited. Practically, several studies have identified EDTA as a calcium chelator, highlighting several concerns for clot formation and wound healing [80]. In vivo studies are necessary to further evaluate the effect that EDTA has on this process. An in vitro study by Balderrama et al. found that dual therapy consisting of decontamination with EDTA followed by a chlorhexidine rinse resulted in impairment of osteoblast adhesion and proliferation [81]. In regard to its decontamination ability, Henderson et al. found that there was no significant difference in the reduction in bacterial load after the use of EDTA when compared with a sterile saline rinse [82].

#### Ethylenediaminetetraacetic Acid (EDTA) Decontamination and Surface Wettability

Kotsakis et al. investigated the effects of various chemotherapeutic agents, including chlorhexidine, citric acid, a 24% EDTA/1.5% NaOCl solution, and sterile saline, on the surface properties and cytocompatibility of titanium implants in the treatment of peri-implantitis. The study demonstrated a reduction in bacterial counts across all the treated surfaces. Notably, surfaces treated with EDTA and NaOCl showed significantly enhanced wettability, with water contact angles reduced to as low as 16.5°, indicating improved hydrophilicity [83].

#### 2.2.5. Sodium Hypochlorite (NaOCl) Decontamination

Sodium hypochlorite (NaOCl) decontaminates implant surfaces through several mechanisms. It breaks down proteins in bacterial cell walls and biofilms by oxidation, causing protein denaturation, structural disruption, and cell lysis. NaOCl also reacts with fatty acids in bacterial membranes (saponification), which enhances membrane disruption and increases penetration. Additionally, NaOCl releases hypochlorous acid (HOCl) in solution, which penetrates cell walls and inactivates bacterial enzymes and nucleic acids through chlorination. This compound also disrupts the biofilm structure by breaking down extracellular polymeric substances, making it easier to remove bacterial colonies. The effects of oral rinsing with NaOCL at a concentration of 0.25% were evaluated by Lee at al. In a clinical study, the authors found a reduction in probing depths and bleeding indexes and suggested that the concentration of 0.25% of NaOCL can be used for the treatment of peri-implantitis [84]. A systematic review by Kyaw et al. claimed that comparatively, the use of NaOCl resulted in better decontamination of dental implant healing abutments when compared with mechanical and laser techniques [85]. Similarly, an in vivo study using NaOCl as a chemical treatment decontamination agent prior to regeneration with autogenous bone displayed favorable outcomes; the re-osseointegration rate of 42% was significantly higher than controls rinsed with saline [84]. These properties were also displayed in an additional in vitro study that utilized the combination therapy of NaOCl and electrolytic cleaning to remove soft and hard deposits from the dental implant abutment surface successfully, all without changing its topography [85].

#### Sodium Hypochlorite and Surface Wettability

At moderate concentrations with extended exposure, NaOCl can effectively decontaminate titanium surfaces while preserving the oxide layer, thus maintaining biocompatibility and supporting osseointegration. In an animal study on rats, Kono et al. demonstrated that treating titanium surfaces with 5% NaOCl for 24 h successfully removed carbon contaminants and converted the surfaces to a superhydrophilic state [86]. However, there is a paucity of studies evaluating the impact of NaOCL on implant surface wettability (Table 5).

## 3. Summary and Conclusions

Physical decontamination techniques use physical forces or energy to remove bacterial biofilms and to decontaminate implant surfaces. These methods include lasers, air-polishing, ultraviolet (UV) light, and cold atmospheric plasma. Erbium-doped yttrium–aluminum–garnet (Er-YAG) lasers have shown effectiveness in reducing bacterial loads, promoting cell adhesion, and enhancing wettability, all without significantly altering implant topography. Air-polishing, which directs abrasive particles to the implant surface, effectively removes biofilms with minimal structural damage, although its impact on wettability varies between titanium and zirconia. UV light induces bacterial cell death through DNA disruption, offering a non-invasive decontamination method that maintains implant integrity and increases wettability for both titanium and zirconia. Cold atmospheric plasma, a newer approach, uses ionized gas to generate reactive species that target microbial cells, providing a highly effective, surface-preserving method that also enhances wettability.

Chemical treatment decontamination methods involve applying antimicrobial agents to neutralize bacteria and pathogens on implant surfaces. This review discusses CHX, EDTA, citric acid, hydrogen peroxide, and sodium hypochlorite, each of which have distinct modes of action. EDTA, a chelating agent, disrupts bacterial cell walls by binding the metal ions essential for stability but does not affect wettability. Citric acid, with its antibacterial and biofilm-disrupting properties, effectively removes bacterial deposits without altering the implant structure, although its effect on wettability remains unclear. Hydrogen peroxide disrupts bacterial cell membranes and has been shown to increase the wettability of titanium surfaces. Sodium hypochlorite, a broad-spectrum disinfectant, is effective but requires careful handling to avoid adverse reactions; its impact on wettability is also unclear.

This literature review underscores the importance of physical and chemical surface decontamination methods and their effects on the wettability of titanium and zirconia implant surfaces. The findings reveal conflicting results regarding the impact of various decontamination techniques—such as laser treatment, air-polishing, UV light, plasma, and different chemical agents—on the hydrophilicity of these implants. To enhance our understanding of which methods, or combinations thereof, are most effective for optimizing implant surface treatment and promoting better osseointegration or re-osseointegration during peri-implantitis therapy, further research in this area is essential.

## 4. Challenges and Future Research

While substantial progress has been made in the development of surface treatments for dental implants, several challenges remain. There is wide variation in the specific laser parameters (wavelength, intensity, pulse duration, etc.) and surface types (e.g., rough, smooth, SLA), for example, used across studies. These inconsistent methodologies make it difficult to compare results directly or to draw definitive conclusions about the optimal treatment protocols for decontaminating or enhancing titanium or zirconia surfaces. While surface modifications such as roughness, wettability, and topography are frequently examined, less attention is given to those biological outcomes that are ultimately the most relevant in clinical practice, such as implant osseointegration, soft-tissue healing, and stability of the grafting site for peri-implantitis management. Based on the included in vitro studies, we are able to show how surface modifications have an impact on hydrophilicity. Further clinical studies, a deeper understanding of bacterial adhesion, improved decontamination protocols, and advancements in personalized treatment strategies are needed to optimize the long-term success of implants in relation to the modification of implant surface properties and improvements in implant surface wettability.

## Figures and Tables

**Table 1:** Implant surface decontamination methods.

**Physical Decontamination Methods**
Lasers
Air-polishing
UV Light
Plasma
**Chemical Treatment Decontamination Methods**
Chlorhexidine (CHX)
Hydrogen Peroxide (H_2_O_2_)
Citric Acid
Ethylenediaminetetraacetic Acid (EDTA)
Sodium Hypochlorite (NaOCl)

**Table 2:** Surface decontamination with lasers.

Study: Lasers	Decontamination Method	Impact on Surface Roughness	Impact on Wettability	Biocompatibility/ Biological Effects	Key Findings
Kreisler et al. (2002)	Er:YAG laser	N/A	N/A	N/A	Decontamination of implant surfaces by means of the Er:YAG laser did not excessively heat the periimplant bone.
Friedmann et al. (2006)	Er:YAG laser (23.5 s with a pulsed, water-cooled laser beam)	Er: YAG laser can create micro-textures that enhance the surface roughness.	Wettability is enhanced after laser treatment, promoting osteoblast attachment.	Osteoblasts show greater attachment on laser-treated surfaces.	Er:YAG laser removed *P. gingivalis* and cellular debris, providing a suitable surface for the attachment of new osteoblasts.
Romanos et al. (2006)	CO2 laser (10,600 nm; 4–6 W; 20 Hz, dual cycle 6%) or Er,Cr:YSGG laser (2780 nm; 1.25 W; air 42; water 41)	N/A	N/A	Osteoblasts may be grown on all of the surfaces.	Laser irradiation of titanium surfaces with carbon dioxide or Er,Cr:YSGG lasers may promote osteoblast attachment and osteoblastic proliferation.
Ayobian-Markazi et al. (2015)	Er:YAG laser (10 Hz; 230 μs pulse duration; 100 mJ/pulse; 12.8 J/cm^2^ energy density) with a cone-shaped fiber tip. SLA titanium surfaces.	Decreased surface roughness.	Increased wettability.	Biocompatibility is enhanced, showing better cell adhesion and growth.	Er:YAG laser irradiation (100 mJ/pulse, 10 Hz) enhances the biocompatibility and osteoblast response of SLA titanium surfaces, reducing the surface roughness and increasing the wettability.
Amid et al. (2021)	Er:YAG, (MZ6 tip; 150 mJ/pulse; 1.5 W; 10 Hz frequency). Air-polishing with glycine.	N/A	Er:YAG and air-polishing both increased wettability.	N/A	Air-flow abrasion improved titanium surface characteristics without altering their topography or elements.
Sousa et al. (2022)	Ti brushes (TiB), TiB with photodynamic therapy (λ: 660–675 nm; 11 mW), TiB with 0.2% CHX/1% NaClO, and treatments with or without UV-C radiation.	TiB decreased Ra.	Increased wettability with TiB plus UV-C irradiation.	No treatment hindered titanium biocompatibility; however, chemical agents and micro-rough surfaces increased cytotoxicity in MG-63 cells, while smooth surfaces and TiO2 photofunctionalization improved cytocompatibility post-decontamination.	UV-C photofunctionalization improves Ti surface biocompatibility and MG-63 cell proliferation, with smooth surfaces and TiO_2_ photofunctiona-lization yielding optimal results.
Rezeka et al. (2024)	Diode lasers (940 nm; 1 W, 2W, or 3W). All groups irradiated for 30 s in continuous mode.	Laser irradiation decreased the roughness by melting the surface.	Wettability increased after laser treatment.	Diode lasers (940 nm) at 2 W and 3 W enhanced titanium surface hydrophilicity, potentially improving implant osseointegration after decontamination.	Diode lasers (940 nm) at 2 W and 3 W power significantly altered the surface characteristics of the material.
Al-Khafaji and Hamad (2020)	Laser structuring.	Ra for the dot-and-groove structuring design increased with an increasing number of laser scans. Laser structuring with the dot design for any laser scan caused and increase in Ra.	Wettability of commercial pure titanium disks for the dot-and-groove structuring design increased with an increasing number of laser scans. Laser structuring with the dot design for any laser scan caused increased wettability.	Laser structuring may enhance cell adhesion, including for osteoblasts, due to improved surface roughness and wettability.	Laser dot-and-groove structuring, especially with 25 laser scans, increased the surface roughness and wettability of CP Ti disks.
Khosroshahi et al. (2009)	Nd-YAG laser (1.06 μm wavelength; 200 μs pulse duration and pulse energy of 50 J).	Increased Ra after Nd-YAG laser treatment from 0 J cm^−2^ to 100 J cm^−2^ and decreased Ra after laser radiation at 140 J cm^−2^.	Decreased wettability after laser radiation from 0 J cm^−2^ to 100 J cm^−2^ and increased wettability after laser radiation at 140 J cm^−2^.	Decrease in contact angle due to Nd-YAG laser treatment can cause more cell adhesion to the surface	SEM, contact angle, and preliminary in vitro/in vivo tests show that Nd laser treatment enhances cell adhesion and improves the physicochemical properties of the Ti6Al4V alloy.
Lee et al. (2023)	Er,Cr:YSGG laser (2780 nm; 2 W; 300 mJ; 10 Hz; 10 sec), diode laser (940 nm; 2 W; 100 mJ; 10 Hz; 10 sec), and electrocautery.	Er,Cr:YSGG laser increased the roughness of SLA- and femtosecond-laser-treated disks compared with machined surfaces.	Er,Cr:YSGG laser on femtosecond-laser-treated surfaces showed statistically significant differences compared with other treatment methods.	Increased O2 levels after Er,Cr:YSGG laser (promotes formation of TiO2 layer)	Er,Cr:YSGG laser and electrocautery treatments signifi-cantly altered the surface roughness of titanium implant surfaces.
Staehlike et al. (2022)	Laser structuring and cold atmospheric plasma.	Laser structuring increases Ra.	Laser-induced microstructures are hydrophobic, but they are hydrophilic after argon plasma activation.	Surface nano–microtopography combined with plasma chemistry enhances the strong attachment of human gingival HGF-1 fibroblasts to zirconia surfaces.	Both laser microstructuring and argon plasma activation of zirconia seems to be optimal for strong gingival cell attachment.

**Table 3:** Surface decontamination by air-polishing.

Study	Decontamination Method	Impact on Surface Roughness	Impact on Wettability	Biocompatibility/Biological Effects	Key Findings
Bennani et al. (2015)	Air-polishing with glycine	Treated disks had a higher mean Ra	N/A	88% reduction in the amount of biofilm on the air-polished disks	Air-polishing is effective in biofilm removal, maintaining surface integrity, and increasing the Ra.
Petersilka et al. (2008)	Air-polishing with glycine or sodium bicarbonate	N/A	N/A	No adverse effects on gingival tissues; improved cleaning efficacy	Glycine powder results in less gingival erosion than hand instrumentation or sodium bicarbonate air-polishing.
Cochis et al. (2013)	Air-polishing with glycine or sodium bicarbonate	Increased Ra with sodium bicarbonate; no change with glycine	Increased wettability	Glycine powder effectively inhibits bacterial recolonization on implants within 24 h	Air-polishing with glycine powder is an effective method for plaque removal from dental implants.
Mistretta et al. (2024)	Air-polishing with glycine or erythritol	Increased Ra	Increased wettability after erythritol air-polishing	Erythritol air-polishing could improve the predictability of regenerative grafting techniques.	Erythritol increased the surface wettability of zirconia disks significantly better than glycine air-polishing.
Francis et al. (2023)	Air-polishing with sodium bicarbonate, glycine, erythritol, or calcium carbonate	No change	N/A	All treated surfaces showed minimal bacterial residues compared with untreated ones	Treatment with different air–powders for 20 s did not alter the surface topography of both M and MRS surfaces.
Drago et al. (2014)	Air-polishing with glycine or erythritol–chlorhexidine powders	N/A	N/A	Erythritol–chlorhexidine air-polishing seemed to display stronger activity than glycine against all the microbial strains tested.	Air-polishing with erythritol-chlorhexidine seems to be a valuable alternative to the traditional glycine treatment.
Kim et al. (2023)	Air-polishing with glycine, 0.12% chlorhexidine and Er:YAG laser	N/A	N/A	Enhanced osseointegra-tion and cell attachment in vivo	Air–powder decontamination improved titanium surface properties and promoted osseointegration in a rabbit tibia model.
Toma et al. (2019)	Plastic curettes, air-polishing with glycine and a titanium brush (Ti-Brush^®^)	N/A	N/A	Different treatments influenced soft-tissue healing and implant stability	Air-polishing and Ti-Brush^®^ protocols were more effective than plastic curettes.
Hentenaar et al. (2022)	Air-polishing with erythritol	N/A	N/A	No significant differences between both groups for mean peri-implant log-transformed bacterial counts.	Erythritol air-polishing was not more effective than saline for implant surface cleansing during peri-implantitis surgery.
Huang et al. (2019)	Air-polishing with glycine, titanium curette treatment, carbon-fiber-reinforced plastic curette treatment, ultrasonic scaling with carbon-fiber-tip treatment	No change Curettes on Ti disks increased Ra	Air-polishing treatment groups showed significan-tly reduced hydrophili- city	For titanium disks, curettes were shown to create deep scratches and increased surface roughness, thereby facilitating the adhesion of bacteria.	Zirconia showed superior resistance to damage from all of the cleaning procedures.
Toma et al. (2016)	Plastic curette, air-polishing with glycine, Ti-Brush and implantoplasty	N/A	Increased wettability after implantoplasty. Decrease in the surface wettability of the plastic curette disks. Hydrophobic character of control-, plastic curette-, Perio-Flow- and Ti-Brush-treated surfaces.	All treatments improved cell adhesion and proliferation and promoted a mature osteoblastic phenotype.	Laser and chemical methods were more effective in improving osteoblast attachment and cell viability compared with mechanical decontamination.
Matthes et al. (2017)	Air-polishing with erythritol, cold atmospheric pressure argon plasma, or both	N/A	Increased wettability for all methods	Air-polishing restores cell compatibility with microbially contaminated implant surfaces.	Plaque removal with air-polishing rendered specimen conducive to cell growth.
Kister et al. (2017)	Diamond burs, polishers, plastic and metal hand instruments, air scalers, and air-polishing with sodium bicarbonate	Air-flow device and plastic curette both showed a minor increase in Ra	Increased wettability after use of an air scaler, air-polishing device, and ProCup treatments	Different cleaning instruments affected the nanocoating integrity and biocompatibi-lity.	Air–powder abrasives and plastic instruments did not damage titanium implant surfaces.

**Table 4:** Surface decontamination with UV light or cold atmospheric plasma.

Study	Decontamination Method	Impact on Surface Roughness	Impact on Wettability	Biocompatibility/ Biological Effects	Key Findings
Rupp et al. (2010)	UV irradiation	N/A	- Anatase surfaces became superhydrophilic (contact angle < 5°) after 75 s of UV treatment with a dosage of 1.9 J/cm^2^. - Ti surfaces showed minimal reduction in contact angle (from 65° to ~58°).	- Enhanced superhydrophilicity on anatase surfaces. - Increased hydroxyl group formation and reduced hydrocarbons, improving surface cleanliness. - Effectively decomposed protein layers on anatase under UV.	UV irradiation removed ~76% of the adsorbed human serum albumin (HSA) on anatase but had no effect on Ti.
Park et al. (2011)	UV irradiation	No significant change in surface roughness (Sa: 0.833 ± 0.032 μm for the control samples; 0.854 ± 0.026 μm for the test samples).	Significant increase in hydrophilicity (contact angle: 22.69° ± 4.35° for test samples; 77.43° ± 3.80° for control samples).	Enhanced initial cellular attachment (*p* = 0.004), increased cell proliferation (*p* = 0.009), and higher ALP synthesis (*p* = 0.016 at day 3; *p* = 0.009 at days 7 and 14).	UV irradiation after 24 h improved wettability, leading to enhanced cellular attachment, proliferation, and differentiation without significant changes in the surface roughness or the oxide layer phase. Carbon content decreased after UV treatment.
Wantanabe et al. (2012)	UV irradiation	No significant change in surface roughness.	Increased wettability. Contact angle = 0° (superhydrophi-lic).	Enhanced cell attachment.	UV treatment (2 h at 19 mW/cm^2^ with UV-C and UV-A radiation) promoted superhydrophilicity and improved osteoblast attachment.
Tuna et al. (2015)	UV irradiation	No topographic changes; roughened surfaces remained rough (Zr1-r, Zr2-r), smooth surfaces remained smooth (Zr1-m, Zr2-m).	Significant shift from hydrophobic to hydrophilic. Contact angles decreased to 2.5–14.1° from 56.4–68.8° before treatment.	UV treatment led to a substantial decrease in surface carbon content (43–81%) and increased oxygen (19–45%) and zirconia (9–41%). The UV treatment increased the monoclinic phase in the Zr1 material by 19–25%.	15 min of UV applied on zirconia surfaces changed the surface properties and significantly improved the wettability.
Rutkunas et al. (2022)	UV irradiation	No significant difference in surface roughness (Ra = 0.094 ± 0.027 µm for ZrO-HT; Ra = 0.11 ± 0.036 µm for ZrO-UTML).	Slight increase in contact angle, but no significant difference (F(16) = 3.50; *p* = 0.292).	No significant difference in cytotoxicity; significant material-dependent changes in fibroblast proliferation (F(8) = 9.58; *p* = 0.005).	UV treatment altered cell viability and promoted cell proliferation in a material-dependent manner. - UV photofunctionalization enhanced HGF cell proliferation, particularly in ultra-translucent zirconia at 72 h.
Hui et al. (2020)	Cold atmospheric plasma	No significant alterations to titanium surface features; preserved implant topography.	No reported changes in wettability.	No adverse biological effects; treatments did not compromise implant biocompatibility.	- CAP alone removed 52.10% of the biofilm, while combining CAP with air abrasion (AA) removed 95.32%. - AA and CAP combined yielded the best decontamination results. - CAP alone showed minimal biofilm removal; this requires further investigation for its potential as a standalone treatment.
Flörke et al. (2022)	Cold atmospheric plasma	No significant change in surface roughness; titanium implant topography preserved.	No significant effect on wettability.	No adverse effects on biocompatibility; significant reduction in bacterial load.	- CAP treatment resulted in the lowest bacterial count (1.24 × 10^5^ CFU/mL), which was significantly lower than that of PDT (8.28 × 10^6^ CFU/mL) and PAG (3.14 × 10^6^ CFU/mL). - CAP also showed the highest reduction in live bacteria and superior antimicrobial effectiveness. - Despite a significant reduction in bacterial load, complete decontamination was not achieved.
Martins et al. (2024)	Cold atmospheric plasma	No significant change in surface roughness.	Significant reduction in contact angle from 59 ± 1.8° (untreated) to 14 ± 2.2° (CAP-treated), indicating improved wettability.	- Improved hemocompatibility with faster clot formation (shorter PT and APTT times). - Increased platelet activation, density, and thrombus formation. - Reduced bacterial colony formation by *Pseudomonas aeruginosa*.	CAP treatment enhanced the surface wettability, improved the interaction with blood components, and reduced bacterial colonization, making it a promising method for decontaminating titanium implants while enhancing biocompatibility.

**Table 5:** Surface decontamination with chemical methods.

Study	Decontamination Method	Impact on Surface Roughness	Impact on Wettability	Biocompatibility/Biological Effects	Key Findings
Stuani et al. (2021)	CHX	Decreased roughness.	No significant differences in wettability.	Residual CHX remained on the surface of the titanium implant, even after rinsing.	CHX displayed substantivity on titanium surfaces.
Bayrak et al. (2022)	CHX	No significant change in surface roughness.	Significant increase in wettability.	CHX residue adsorbed onto the titanium SLA surface.	The use of 0.2% CHX solution for 60 s improved surface wettability.
Mouhyi et al. (2000)	H_2_O_2_ + CO_2_ laser	N/A	Significant increase in wettability.	Restoration of the oxide layer onto the titanium implant surface.	A combination of H_2_O_2_ + CO_2_ laser seems effective in the re-establishment of the atomic composition and oxide structure of contaminated titanium surfaces.
Yoneyama et al. (2013)	H_2_O_2_ hydrothermal oxidation	SEM analysis showed increased surface roughness after treatment.	Significant increase in wettability.	Loading with fibroblast growth factor-2 (FGF-2) promoted the initial cell adhesion, proliferation, and osteodifferentiation, and enhanced bone bioactivity.	This treatment increased the initial adhesion, proliferative, and osteodifferentiation capacities of cells on the surface of titanium disks and promoted bone formation around a mini-implant.
Lee et al. (2021)	Brushing implant surfaces with 3% H_2_O_2_	N/A	N/A	This treatment restrained biofilm growth.	Compared with mechanical debridement, brushing implant surfaces with 3% H_2_O_2_ superiorly restrained biofilm regrowth.
Noro et al. (2013)	Immersion in 150 mM H_2_O_2_ solution at 60 °C for 1 day	N/A	No significant differences in wettability on zirconia surfaces.	Introduction of hydroxyl groups onto zirconia surfaces can decrease the carbon content and therefore improve wettability.	No significant differences in wettability on zirconia surfaces.
Tuna et al. (2023)	Immersion in 20% H_2_O_2_ for two hours	No significant change in surface roughness.	Significant increase in wettability.	Treatment with H_2_O_2_ appears to have no effect on the mineralization capacity of osteoblasts. However, the risk of thermal degradation of zirconia polycrystals does increase.	H_2_O_2_ treatment increases wettability and has no influence on osteoblast behavior. However, this treatment can be detrimental to material stability.
Wheelis et al. (2016)	40% citric acid	Significant increase in surface roughness	N/A	40% citric acid is corrosive and disrupts the oxide layer on the titanium implant. The authors assume this has a negative effect on wettability.	While no investigation of wettability was performed, the authors assume that disruption of the oxide layer will hinder re-osseointegration.
Souza et al. (2019)	Citric acid	Increased surface roughness.	N/A	Treatment significantly hindered the re-colonization of bacterial biofilms.	No effect on the oxide layer was observed. The treatment statistically enhanced the electrochemical stability of the titanium disk.
Kotsakis et al. (2016)	24% EDTA/1.5% NaOCl solution	N/A; the analysis only mentioned that the treatment left elemental surface contaminants.	Significant increase in surface wettability.	A significant increase in osteoblast proliferation was observed compared with surfaces decontaminated with CHX.	Osteoblast proliferation and differentiation did not significantly differ between decontaminated surfaces and controls.
Kono et al. (2015)	5% NaOCl for 24 h	No significant changes in surface roughness.	Significant increase in surface wettability.	Biomechanical push-in test indicated that the bone–titanium integration strength was significantly stronger in decontaminated surfaces.	NaOCl pretreatment enhanced the osseointegration capability of titanium and converted the surfaces from hydrophobic to superhydrophilic.

## Data Availability

No new data were created or analyzed in this study. Data sharing is not applicable to this article.
